# Supplementary material for: The impact of clergy sexual abuse on spirituality and health: A systematic scoping review of the literature
Source: PLoS One. 2025 Apr 16;20(4):e0317821. doi: 10.1371/journal.pone.0317821 (PMC12002452; doi:10.1371/journal.pone.0317821)
Supplement: S2 File — (DOCX) [file pone.0317821.s002.docx]

| **Databases Search**  **How** | **Date of Search** | **Search String** | **Limiters** | **Returns/ Search Results** | **Action Taken** | **Database information** |
| --- | --- | --- | --- | --- | --- | --- |
| **Academic Search Complete va EBSCO Host; University of New England Armidale Library** | 23 November 2022  Follow up search  November 2023 | (TI Clerical OR Clergy OR Church OR Religio* OR Cathol* AND AB Clerical OR Clergy OR Church OR Religio* OR Cathol*) AND (**S1 AND S2 AND S3**)  **S1**  TI ( Spiritual* or Religio* or devout ) AND AB ( Spiritual* or Religio* or devout ) **S2**  TI ( Harm OR Abuse OR Trauma* OR damage ) AND AB ( Harm OR Abuse OR Trauma* OR damage )  **S3**  TI (Clerical OR Clergy OR Church OR Religio* OR Cathol*) AND AB (Clerical OR Clergy OR Church OR Religio* OR Cathol* ) | Date 2002 –  2022  Follow up search  Date 2022-2023 | 252  239 with duplicates removed | Exported to Endnote 23 November 2022  Duplicates  239 with duplicates removed |  |
| **Informit Database**  **University of New England Armidale Library** | 23 November 2022  Follow up search  November 2023 | [All Fields: clerical OR All Fields: clergy OR All Fields: church OR All Fields: religion] AND [All Fields: harm OR All Fields: abuse OR All Fields: trauma OR All Fields: damage] AND All Fields: spiritual or religion AND Resource Type: Journal AND Publication Date: (01/01/2002 TO 12/31/2022) | Date: 01/01/2002 TO 12/31/2022  Resource Type: Journal  Follow up search  Date 2022-2023 | 51 | Exported to Endnote 23 November 2022  Duplicates  12 removed  39 with duplicates removed |  |
| **Web of Science** | 23 November 2022  Follow up search  November 2023 | **Clerical OR Clergy OR Church OR Religio* OR Cathol*** (Title) and **Spiritual* or Religio* or devout** (Title) and **Harm OR Abuse OR Trauma* OR damage** (Title)  AND  **Clerical OR Clergy OR Church OR Religio* OR Cathol*** (All Fields) and **Spiritual* or Religio* or devout** (All Fields) and **Harm OR Abuse OR Trauma* OR damage** (All Fields)  Results link  <https://www.webofscience.com/wos/woscc/summary/17d14ee2-2e9c-40b9-819b-3f30d4b2bd70-60601062/relevance/1> | Date  2002-01-01 to 2022-01-01 (Publication Date)  Article (Document Types) and English (Languages) and Article (Document Types)  Follow up search  Date 2022-2023 | 226 | Exported to Endnote 23 November 2022 |  |
| **ProQuest** | 23 November 2022  Follow up search  November 2023 | **title(Spiritual* OR Religio* OR devout) AND title(Harm OR Abuse OR Trauma* OR damage) AND title(Clerical OR Clergy OR Church OR Religio* OR Cathol*)**  **Limits applied**  **Search Link**  **https://www.proquest.com/search/2266011?accountid=17227** | Scholarly Journals OR Dissertations & Theses  Article OR Dissertation/Thesis OR Review OR Editorial  Dates: 2002 – 2022  English  Follow up search  Date 2022-2023 | 806 | Exported to Endnote 23 November 2022  Removed duplicates with endnote and then manually  282 with dups removed |  |
| **Google Scholar** | 23 November 2022  Follow up search  November 2023 | **(Spiritual* OR Religio* OR devout) AND (Harm OR Abuse OR Trauma* OR damage) AND (Clerical OR Clergy OR Church OR Religio* OR Cathol*)**  **Second search**  **"Spiritual Harm" "Spiritual Abuse" "Spiritual Trauma"** | Dates: 2002 – 2022  Screened first 10 pages  Follow up search  Date 2022-2023 | Returns 26,700  Reviewed possible  21  Exported:  6 |  |  |
