# Supplementary material for: The impact of clergy sexual abuse on spirituality and health: A systematic scoping review of the literature
Source: PLoS One. 2025 Apr 16;20(4):e0317821. doi: 10.1371/journal.pone.0317821 (PMC12002452; doi:10.1371/journal.pone.0317821)
Supplement: S3 File — (DOCX) [file pone.0317821.s003.docx]

| **NO** | **Author/**  **Year** | **Country** | **Title** | **Concept focus** | **Impact** | **Conceptual Note** | **Act of Abuse/perpetration** |
| --- | --- | --- | --- | --- | --- | --- | --- |
| 1 | Benkert & Doyle (2009) | **United States of America** | Clericalism, Religious Duress and its Psychological Impact on Victims of Clergy Sexual Abuse. | Religious duress | Can seriously impede a person’s capacity to accurately perceive and evaluate abusive actions perpetrated on them by clergy  Insidious and unrelenting in its destruction, causing irreversible wounds. |  |  |
| 2 | Bland (2002) | **United States of America** | The psychological and spiritual effects of child sexual abuse when the perpetrator is a Catholic priest. | Spiritual Injury | Negative psychological and spiritual long-term effect  Inability to draw on religion or spirituality as a coping strategy against the stress of adverse life changes or events |  |  |
| 3 | Doyle  (2009) | **United States of America** | The Spiritual Trauma Experienced by Victims of Sexual Abuse by Catholic Clergy. | Spiritual Trauma | The spiritual pain suffered by one who feels cut off or abandoned translates into depression or, in its extreme, despondence. Often there is a significant amount of anxiety that gradually turns to depression. The abused person continually encounters situations that require some form of spiritual support such as deaths, births, illness or loss. The spiritual support always came from the external symbols or from the priest or minister to whom he or she turned for support and guidance.  The natural reaction to turn to the Church or a priest is met by a psychological or emotional reaction derived from the abuse. The source of security is now a source of pain. The frustration and anxiety are grounded in the perceived futility from seeking a source of spiritual assistance and finding none. | Seminal text on Spiritual Trauma and should be read in full |  |
| 4 | Ellis et al.,  (2022) | **United States of America** | Religious/spiritual abuse and trauma: A systematic review of the empirical literature. | Religious / Spiritual Abuse and Trauma | Psychological Harm; inducing negative psychological impact; Having a negative impact on one’s spiritual health. | Johnson and Van Vonderen (1991) deﬁne Religious/Spiritual abuse as “the mistreatment of a person who is in need of help, support, or greater spiritual empowerment, with the result of weakening, undermining, or decreasing that person’s spiritual empowerment” (p. 23).  Review found that Religious/Spiritual abuse and trauma was deﬁned in a variety of ways across 25 studies | Misuse of Power or using a God or higher power to control or induce fear in others; with the goal of coercing, controlling, or exploiting another person. |
| **5** | Farrell,  (2009) | **United Kingdom** | Sexual abuse perpetrated by Roman Catholic priests and religious. | Theological Trauma | **Theological conflict ; Significant harm to spiritual identity**  **Existentialism, sense making, shattering of worldview and safety within the world; Being in conflict with God**  they can be re-traumatized if they disclose abuse to family and religious community, particularly if the response is not supportive  deep anger towards their abuser and the Church are core emotional components; Feeling of abandonment; robbed of spiritualality/faith and fundamental philosophy for living. | Difficulty praying  Discomfort with religion assuming ownership of the spirit  Generalized sense of inner emptiness  Locked into continual conflict with God  Inability to engage in any of the sacraments  Political anger | …clerical role including that of community trust, charisma, patriarchal privilege, and power…unique to the Roman Catholic priest, and that they are God’s representative on earth. Their role in a community is therefore profoundly different. |
| **NO** | **Author/**  **Year** | **Country** | **Title** | **Concept focus** | **Impact** | **Conceptual Note** | **Act of Abuse/perpetration** |
| 6 | Guido, (2008) | **United States of America** | A Unique Betrayal: Clergy Sexual Abuse in the Context of the Catholic Religious Tradition. | Spiritual and religious abuse | Danny hoped that his story might help others. “Tell them,” he insisted, “what he took away from me. Not just my innocence but my faith. I’m like a spiritual orphan, betrayed by what I loved, and I feel lost and alone.”  Such desecration can pertain to the sacredness of the victim, whose body, mind and faith are violated; the priesthood, the expectations of which are betrayed; the Church, which is viewed as complicit; and God, whose goodness, protection and very existence may be called into question.  In this sense, what makes clerical abuse unique is not so much who perpetrates the abuse but rather its implications for an individual's (or congregation's) relationship to the unique domain of the sacred. |  | Spiritual and religious abuse entails a desecration of the sacred both in itself (spirituality) and in terms of its meaning and significance (religion). |
| 7 | McPhillips (2018a) | **Australia** | Silence, Secrecy and Power: Understanding the Royal Commission Findings into the Failure of Religious Organisations to Protect | Spiritual Harm | The Royal Commission identified theology as  a key characteristic in facilitating the sexual abuse of children. Perpetrators commonly manipulated images of God, and threatened children with spiritual harm. In Catholic and Anglican churches, children were abused in spiritual practices such as confession and mass preparation where the cleric could be alone with the child (Royal Commission 2017b: 52). Religious rituals, symbols and language were used to manipulate and confuse children. This caused spiritual harm and led to many victims abandoning their faith and losing their trust in the religious organisation (Royal Commission 2017b: 52; McPhillips 2018)  That spiritual trauma was a common result of sexual abuse and had serious impacts on the well-being and agency of survivors McPhillips (2018b)  Feeling a loss of trust in institutions and others is also reported (Doyle, 2009) |  |  |
| 8 | Novsak et al.,  **(2012)** | **Slovenia** | Therapeutic implications of religious-related emotional abuse. | Religious related emotional abuse | Causing harm our relationship with God and/or separate us from him (God). | Multifaceted issue | Occasions when religion or certain religious beliefs correlate with various types of abuse.  Religious abuse includes a range of actions by pastors, counselors, and other people that harm our relationship with God and/or separate us from him (Cumella, 2005). |
| **NO** | **Author/**  **Year** | **Country** | **Title** | **Concept focus** | **Impact** | **Conceptual Note** | **Act of Abuse/perpetration** |
| 9 | Pargament et al., (2008) | **United States of America** | Problem and Solution: The Spiritual Dimension of Clergy Sexual Abuse and its Impact on Survivors. | Spiritual Dimension of Clergy Sexual Abuse | Profound negative spiritual effect on individual’s relationship with Church and God  Loss of religion and faith; Anger at God, Jesus and the Church |  |  |
| 10 | Spraitz & Bowen (2020) | **United States of America** | Religious Duress and Reverential Fear in Clergy Sexual Abuse Cases: Examination of Victims’ Reports and Recommendations for Change. | Religious Duress and Reverential Fear |  |  | Religious duress is similar to the idea of reverential fear, which intensifies based on the high levels of respect that one has for a person in position of authority. |
| 11 | Pereda & Segura (2021) | **Spain** | Child Sexual Abuse Within the Roman Catholic Church in Spain: A Descriptive Study of Abuse Characteristics, Victims' Faith, and Spirituality. | Spiritual damage | Experience of CSA by a representative of the Church had an extreme or considerable impact on their belief in the Catholic Church and in God.  Spiritual discontent/Trauma |  |  |
| 12 | McPhillips (2018b) | **Australia** | “Soul Murder”: Investigating Spiritual Trauma at  the Royal Commission | Spiritual Trauma | A complex relationship between spirituality, religion and sexual trauma with variable outcomes, including significant levels of spiritual injury.  Spiritual trauma is associated with the following: a loss of the spiritual dimensions of self-identity; damage to the ability to construct meaning; and “a conflicted or broken relationship with God, a loss of trust in religious institutions, and an impaired ability to develop spiritually”(Pargament et al., 2008) | A subset of a wider field of psychosocial  research concerned with spirituality, religion and trauma |  |
