# Supplementary material for: The impact of clergy sexual abuse on spirituality and health: A systematic scoping review of the literature
Source: PLoS One. 2025 Apr 16;20(4):e0317821. doi: 10.1371/journal.pone.0317821 (PMC12002452; doi:10.1371/journal.pone.0317821)
Supplement: Attachment — Submitted filename: Response to Reviewers PLOS One Scoping Review 191124.docx [file pone.0317821.s005.docx]

To the editorial team and the reviewers,

RE: Response to Reviewers

Thank you for considering our manuscript for publication. We have reviewed them carefully and provided revisions to the original manuscript based on your feedback. I have tabled all comments individually and provided an additional response column to demonstrate the changes we have made.

We have also uploaded the document 'Revised Manuscript with Track Changes’ which outlines all changes made to the original submission. An unmarked version labelled 'Manuscript' is also provided as requested.

| Editorial and Peer Review Comments | Response and Change |
| --- | --- |
| Thank you for stating the following financial disclosure:  “Health Equity Grant St Vincent's Health Australia “ Please state what role the funders took in the study.  If the funders had no role, please state: "The funders had no role in study design, data collection and analysis, decision to publish, or preparation of the manuscript."  If this statement is not correct you must amend it as needed.  Please include this amended Role of Funder statement in your cover letter; we will change the online submission form on your behalf. | **Thank you. This is useful. The section has been added to the funding section**  ***The funders had no role in study design, data collection and analysis, decision to publish, or preparation of the manuscript*** |
| We note that you have referenced (Unpublished Ph.D. Thesis) on page 25, which has currently not yet been accepted for publication. Please remove this from your References and amend this to state in the body of your manuscript: (ie “Bewick et al. [Unpublished]”) as detailed online in our guide for authors [http://journals.plos.org/plosone/s/submission-guidelines#loc-reference-style](https://urldefense.com/v3/__http:/journals.plos.org/plosone/s/submission-guidelines*loc-reference-style__;Iw!!LUsMDrd6!m82Pgd_I3Lq_C9GG8q78DHKb8iMZUi78Q1bBApFlXaDAId1IYWYtL55krhya8JvDIN_wwXl0YG7Bb4YUo2U7$) | Thank you. I have amended the reference and now listed two citations – one for the paper which lists the use of the PTSD-QAI (Farrell D. Sexual abuse perpetrated by Roman Catholic priests and religious. Mental Health, Religion & Culture. 2009;12(1):39-53.) and the other which is the unpublished thesis. This now states that the thesis is unpublished in the reference list rather than refer to a specific date. |
| Please include captions for your Supporting Information files at the end of your manuscript, and update any in-text citations to match accordingly. Please see our Supporting Information guidelines for more information: [http://journals.plos.org/plosone/s/supporting-information](https://urldefense.com/v3/__http:/journals.plos.org/plosone/s/supporting-information__;!!LUsMDrd6!m82Pgd_I3Lq_C9GG8q78DHKb8iMZUi78Q1bBApFlXaDAId1IYWYtL55krhya8JvDIN_wwXl0YG7Bbzranx9L$). | Thank you. All summary files have titles and have been listed at the end of the manuscript as instructed. |
| Comments from the Editorial Office: Please justify your choice for focusing this study on the Catholic church.  [Note: HTML markup is below. Please do not edit.] | Thank you, we have strengthened this justification in the introduction and the background and focus of the review by both adding citations to justify the focus and also adding additional text which highlights the particularity of the harm in the Catholic Church context.  These changes are highlighted in the tracked change manuscript. |
| 1. Is the manuscript technically sound, and do the data support the conclusions?  The manuscript must describe a technically sound piece of scientific research with data that supports the conclusions. Experiments must have been conducted rigorously, with appropriate controls, replication, and sample sizes. The conclusions must be drawn appropriately based on the data presented.  Reviewer #1: Yes  Reviewer #2: Yes  Reviewer #3: Yes  Reviewer #4: Partly | Thank you |
| . Has the statistical analysis been performed appropriately and rigorously?  Reviewer #1: Yes  Reviewer #2: Yes  Reviewer #3: N/A  Reviewer #4: Yes | Thank you |
| Have the authors made all data underlying the findings in their manuscript fully available?  The [PLOS Data policy](https://urldefense.com/v3/__http:/track.editorialmanager.com/CL0/http:*2F*2Fwww.plosone.org*2Fstatic*2Fpolicies.action*23sharing/1/010f01930c6e556c-97143587-2fa2-4b60-8f74-68e5ac6afb69-000000/8YmCwv5jKXaV7PKvv8RMc92bS2x0x25IadvLXncGfZo=184__;JSUlJSU!!LUsMDrd6!m82Pgd_I3Lq_C9GG8q78DHKb8iMZUi78Q1bBApFlXaDAId1IYWYtL55krhya8JvDIN_wwXl0YG7BbzxsIURA$) requires authors to make all data underlying the findings described in their manuscript fully available without restriction, with rare exception (please refer to the Data Availability Statement in the manuscript PDF file). The data should be provided as part of the manuscript or its supporting information, or deposited to a public repository. For example, in addition to summary statistics, the data points behind means, medians and variance measures should be available. If there are restrictions on publicly sharing data—e.g. participant privacy or use of data from a third party—those must be specified.  Reviewer #1: Yes  Reviewer #2: Yes  Reviewer #3: Yes  Reviewer #4: Yes | Thank you |
| Is the manuscript presented in an intelligible fashion and written in standard English?  PLOS ONE does not copyedit accepted manuscripts, so the language in submitted articles must be clear, correct, and unambiguous. Any typographical or grammatical errors should be corrected at revision, so please note any specific errors here.  Reviewer #1: Yes  Reviewer #2: Yes  Reviewer #3: Yes  Reviewer #4: Yes | Thank you |
| Reviewer #1: The author has provided an examination of scientific literature on the topic of spiritual harm, explaining the concept and its relevant measures. The paper is well designed, it indicates the complexity of the analyzed theoretical approach. | Thank you. |
| However, the section DISCUSSION does not present well the main findings elaborated in this study.  It has to be more detailed showing better the connections between the key concepts introduced in this study. | Thank you. We have been through the discussion and made these points clearer. The discussion now better connects to the key concepts in the study. |
| Reviewer #3: Dear authors,  This is an excellent and rigorous systematic scoping review. It was very clear and easy to follow the steps that you had taken in understanding how Spiritual Harm is understood in contemporary academic journals. You do well to recognise the limitations and offer future research direction. It is technically sound and the steps are clear to replicate. | Thank you |
| My recommendation is to accept, but there are two points that I am not clear on relating to 4. Positionality. The first is to offer a better explanation for what "...some understand the harm perpetrated through their research, support and/or advocacy for victims and survivors" means. I was not clear on what this harm looks like through the research that you do. | Thank you. We agree this wasn’t clear and have amended to read as:  *Some authors have lived experienced of sexual abuse in a Church context as survivors and some do not have a lived experience of the harm, but support survivors of sexual assault through their research, and/or advocacy for victims and survivors.* |
| The second, you discuss at the end that you had open dialogue about your own experiences which is very important and you considered the influence, but my question is what did this influence have on your own individual understandings but also the larger decisions made regarding the reporting of the results. | *Thank you.*  *We have strengthened this section and revised as follows:*  *Throughout this review, all authors had open dialogue about their professional, personal and spiritual experiences and considered the influence this had on understanding and decisions regarding the reporting of results. These conversations helped us to understand potential biases and blind spots we could encounter prior to undertaking the scoping review. This allowed us to ensure all biases were managed and avoided. Collectively, the authors agreed to adhere to the original protocol, clearly and transparently document all processes undertaken in order to minimise the potential for undue influence based in personal perspectives. The authors took every precaution to ensure that personal perspectives did not have an undue influence on the reporting of results.* |
| On p.16 I found a double comma. Under background: "Comprised by" could be "comprised of". Otherwise, it is very well written. | Comma removed and comprised of changed |
| Reviewer #4: The present manuscript The impact of clergy sexual abuse on spirituality and health: a systematic scoping review of the literature shows and details a quality scientific research contemplating all the components to be considered in order to publish in this journal.  The search strategies implemented to support the research are adequate and are carefully detailed in the development of this manuscript. The three stages executed as part of the strategies are convenient and allow achieving the proposed objectives, and the data clearly show a systematic review of studies on the subject. | Thank you this is useful. |
| The conclusions should be addressed with greater precision and correspond to the central objective of the project for which this research has taken place, which the authors of the manuscript state is to address the harm experienced by survivors of sexual abuse in the Church in contexts of health and spiritual care, in this sense a greater forcefulness is lacking in the conclusions which do not allow contributing relevant information to the project in question. | Thank you. We have revised the discussion and strengthened this section to make the conclusions clearer. We appreciate this feedback. |
| The manuscript has been written in English, it is adequate for the parameters of the journal, the language used is simple and at the same time respects the parameters of spelling and writing. | Thank you |
